# Blunt Trauma to the Neck Presenting as Dysphonia and Dysphagia in a Healthy Young Woman; A Rare Case of Traumatic Laryngocele

**DOI:** 10.30476/BEAT.2020.46455

**Published:** 2020-04

**Authors:** Saptarshi Biswas, Manick Saran

**Affiliations:** 1 *Department of Trauma and Acute Care Surgery, Forbes Hospital, Allegheny Health Network, Pennsylvania, USA*; 2 *Lake Erie College of Osteopathic Medicine(LECOM), Erie, Pennsylvania, USA*

**Keywords:** Traumatic laryngocele, Blunt trauma, Conservative management, Resolution

## Abstract

Laryngocele is not a common clinical entity that presents itself in a trauma setting. In the literature, there are currently two types of laryngocele, internal and mixed. Laryngocele may be congenital or acquired, and most often will present later in life. Traumatic laryngocele has only been reported three times in the literature before. Herein, we report a rare case of a 22-year-old woman who presents with bilateral laryngocele secondary to sustained direct trauma. Neck Ct-scan revealed bilateral laryngocele being responsible for her dysphagia and dysphonia. She was monitored in the hospital for further exacerbation of her symptoms with feared airway occlusion in mind. On hospital day three, her dysphagia had resolved and her dysphonia had significantly improved. A second CT, revealed resolution of left laryngocele with the right decreased in size since the initial presentation. She was followed and had complete resolution of symptoms one week after the injury.

## Introduction

Laryngocele is a rare, benign cyst of the ventricular folds in the larynx occurring in only in one per 2.5 million people [1]. These lesions are abnormal dilations of the laryngeal ventricle that are most often filled with air, or in severe cases may be filled with pus and thus termed laryngopyocele. Currently, the two types of laryngoceles described in modern literature are internal laryngocele and mixed type laryngocele [2]. Several etiologies of laryngocele have been found in a review of the literature, including congenital, increased intralaryngeal pressure, and mechanical obstruction. Laryngoceles are most often asymptomatic and found incidentally on imaging. Traumatic laryngocele has only been reported in the literature three times before [3].

We herein present a rare case of bilateral internal laryngocele caused by direct trauma to the neck of a 22-year-old woman presenting with dysphonia and dysphagia. The onset of dysphagia and dysphonia in a patient after suffering trauma to the neck is a cause for concern, and thus this unique presentation of laryngocele is an important piece of academic discussion. 

## Case report

**Fig. 1:**
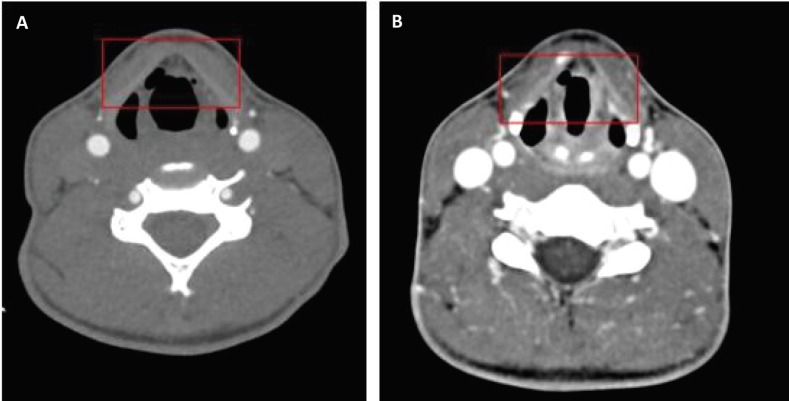
Axial Ct-scan of the neck demonstrating bilateral laryngoceles in the pre-epiglottic space with the right laryngocele larger than the left (A); axial CT-scan of the neck on day 3, demonstrating resolution of left laryngocele with the right decreased in size since the initial presentation

A 22-year-old woman presented to the Emergency Department (ED) as an activated level two trauma. She was the rider of a motorcycle when she lost control resulting in direct trauma to her neck. She was worked up in routine advanced trauma life support (ATLS) protocol. Her primary survey was essentially unremarkable. Notably, she denied any loss of consciousness, her airway was patent and she did not have any stridor on exam. Her secondary survey showed tenderness to palpation of the anterior portion to the left side of her neck. She also complained of muffled voice and dysphagia. The vitals were within normal limits, with her blood pressure at 131/70, heart rate at 90 beats per minute, and her oxygen saturation at 100%. Because of the mechanism of injury, a trauma pan CT-scan was performed. A CT of her head showed no acute intracranial hemorrhage. CT cervical spine and CT-angiography of the neck were essentially normal. CT scan of her neck, however, showed bilateral laryngoceles in her pre-epiglottic space with the right laryngocele larger than the left (Figure 1A). ENT service was consulted and a bedside laryngoscopy was performed. The findings of the scope correlated with the findings of the CT. On the right in her piriform sinus, she had a non-swelling hematoma extending to the posterior pharyngeal wall. The rest of her exam including oropharynx, epiglottis, true vocal cords, anterior and posterior commissure, and arytenoid cartilages was normal. It was determined that the laryngocele noted on CT and through the scope were responsible for her dysphagia and dysphonia. She was monitored in the hospital for further exacerbation of her symptoms with feared airway occlusion in mind. On hospital day three, her dysphagia had resolved and her dysphonia had significantly improved. A second CT, seen in Figure 1B, was done which showed resolution of left laryngocele with the right decreased in size since the initial presentation. She was followed up as an outpatient and had complete resolution of her symptoms one week after the initial injury. 

## Discussion

Currently, laryngocele is reported to affect roughly 1% of the population [1], with men being more likely to be affected than women [3]. Laryngocele may present in any age range, from neonates to incidental findings in the elderly, and can present either a unilateral or bilateral manifestation [4]. However, the currently reported incidence is the sixth decade of life [3]. The relative incidence may actually be much higher due to the asymptomatic nature of these cystic dilations. Laryngocele is most often congenital in nature yet is not uncommon to present when in adulthood. Laryngocele most often presents unilaterally in about 85% of cases, with the rest being bilateral [5]. Adult patients at highest risk for development of laryngocele are those that have congenitally dilated saccules [6]. The saccule is an anatomic region that begins in the laryngeal ventricle and continues superiorly into the connective tissue of the pre-epiglottic space thus explaining why laryngoceles communicate with the laryngeal lumen. Repeated periods of increased intra-glottic pressure [7] such as glassblowers, horn players, defecating, or carcinoma obstructing the lumen are some of the reported hypothetical etiologies of laryngocele. It is well known that during a Valsalva maneuver, both true and false vocal folds close thus increasing intralaryngeal air below the folds [8]. This increased intralaryngeal pressure is hypothesized to further exacerbate the congenitally dilated saccule to result in a laryngocele [9]. As noted above, it has only been reported secondary to direct neck trauma once before in the literature. 

Laryngocele is defined historically as internal, external or mixed. However, recent literature has described only internal, those laryngoceles that are medial to the thyrohyoid membrane and mixed, those laryngoceles that pierce through the thyrohyoid membrane [10]. Internal Laryngoceles present with hoarseness, snoring, upper airway obstruction, globus sensation, and dysphagia with hoarseness and neck swelling being the most common [11]. Diagnostically, most often these are found on incidental imaging or can be diagnosed clinically combined with imaging in patients that are symptomatic. Currently, the most accurate and best imaging modality for the diagnosis of laryngocele is CT imaging [12]. CT imaging of laryngocele will often appear as an air-filled structure in the space near the laryngeal saccule. If underlying squamous cell carcinoma is thought to be the causative factor for the laryngocele, MRI is excellent in showing soft tissue extent as well as to stage the tumor [11].

Smaller internal laryngoceles are managed via microlaryngoscopy with a CO2 laser. However, for larger laryngoceles or those that are mixed in nature, surgeons use an approach through the lateral neck. This method is preferred due to superior exposure and a low recurrence rate. However, higher morbidity, longer surgery, and longer recovery time in the hospital with subsequently increased cost are the disadvantages to this approach [2]. Complications seen in this approach are airway compromise secondary to edema, laryngocutaneous fistula, subcutaneous emphysema, and nerve injury.

External approaches to management of laryngocele have also been described in the literature. The transthyroid membrane approach, thyrotomy with thyroid cartilage resection, and a V-shaped thyrotomy are three procedures that will be discussed here. Both the thyrotomy with cartilage resection and the V-shaped thyrotomy provide superior visualization of the paraglottic space [13] over the transthyroid membrane approach. 

In our patient, the laryngocele was diagnosed incidentally on imaging as part of the trauma workup following a direct injury to her neck. It is quite possible that our patient, based on the literature review described in this case report, had a congenitally dilated saccule that expanded upon trauma to the neck. Her symptoms improved without any intervention.

In conclusion, laryngocele often present asymptomatically, incidentally on imaging or in a patient presenting as ours did with dysphagia and dysphonia. Due to the danger of possible airway compromise or underlying malignancy, laryngoceles, an uncommon entity that must be investigated further. 

## Conflict of Interest:

The authors declare that there is no actual or potential conflict of interest in relation to this paper.
